# Predictive performance of radiomic models based on features extracted from pretrained deep networks

**DOI:** 10.1186/s13244-022-01328-y

**Published:** 2022-12-09

**Authors:** Aydin Demircioğlu

**Affiliations:** Institute of Diagnostic and Interventional Radiology and Neuroradiology, University Hospital Essen, Hufelandstraße 55, 45147 Essen, Germany

**Keywords:** Deep learning, Radiomics, Benchmarking, Machine learning, Radiology

## Abstract

**Objectives:**

In radiomics, generic texture and morphological features are often used for modeling. Recently, features extracted from pretrained deep networks have been used as an alternative. However, extracting deep features involves several decisions, and it is unclear how these affect the resulting models. Therefore, in this study, we considered the influence of such choices on the predictive performance.

**Methods:**

On ten publicly available radiomic datasets, models were trained using feature sets that differed in terms of the utilized network architecture, the layer of feature extraction, the used set of slices, the use of segmentation, and the aggregation method. The influence of these choices on the predictive performance was measured using a linear mixed model. In addition, models with generic features were trained and compared in terms of predictive performance and correlation.

**Results:**

No single choice consistently led to the best-performing models. In the mixed model, the choice of architecture (AUC + 0.016; *p* < 0.001), the level of feature extraction (AUC + 0.016; *p* < 0.001), and using all slices (AUC + 0.023; *p* < 0.001) were highly significant; using the segmentation had a lower influence (AUC + 0.011; *p* = 0.023), while the aggregation method was insignificant (*p* = 0.774). Models based on deep features were not significantly better than those based on generic features (*p* > 0.05 on all datasets). Deep feature sets correlated moderately with each other (*r* = 0.4), in contrast to generic feature sets (*r* = 0.89).

**Conclusions:**

Different choices have a significant effect on the predictive performance of the resulting models; however, for the highest performance, these choices should be optimized during cross-validation.

**Supplementary Information:**

The online version contains supplementary material available at 10.1186/s13244-022-01328-y.

## Key points

- Extraction of deep features entails several decisions influencing the predictive performance.
- Models built with pretrained deep features did not outperform generic models.
- Correlations between pretrained deep and generic feature sets were rather moderate (*r* = 0.43).

**Supplementary Information:**

The online version contains supplementary material available at 10.1186/s13244-022-01328-y.

## Background

Radiomics can be outlined as the automation of the extraction of quantitative data from radiological imaging to support medical tasks such as diagnosis and prognosis. Although such an approach was already spelled out in the late 1970s [1], it became prominent only when it was introduced in a seminal paper by Lambin et al. [2].

**Fig. 1:**
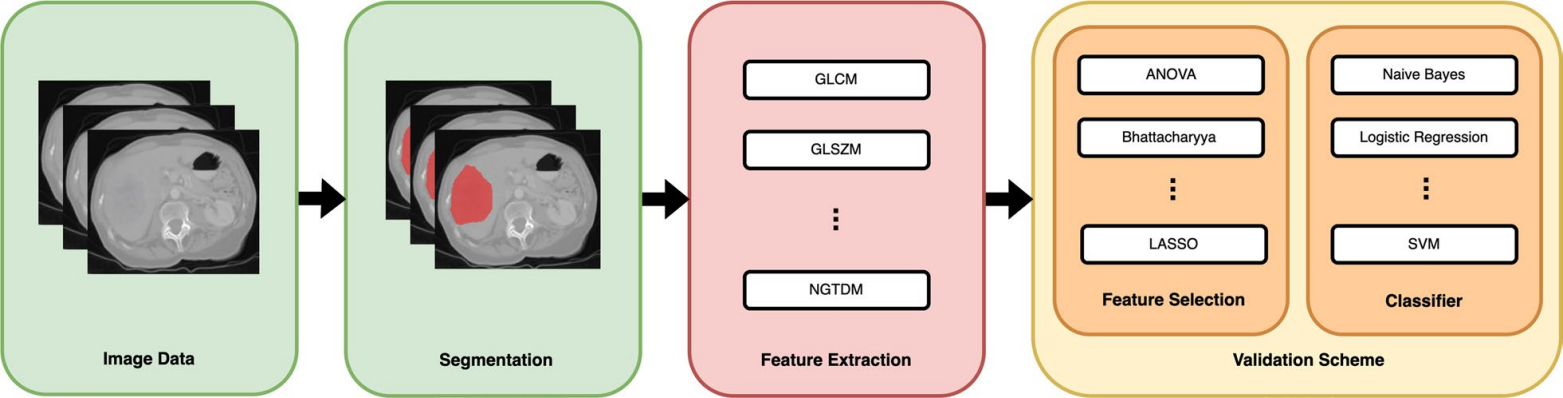
General radiomics pipeline

Analogous to a classical machine learning pipeline (Fig. 1), radiomics proceeds in several steps [3]. A key issue is the generation of features since these need to extract the information in the data. In the radiomics approach, one uses almost exclusively generic morphological and texture features (like diameter and intensity variance) since they are predictive in the context of oncology [4–6]. Even though these features have proven very useful for building highly predictive models [7–9], they are generic, not tailored to a specific problem, and, therefore, suboptimal.

Deep learning (DL), a subset of machine learning based on neural networks, has recently been successfully applied to various classification tasks [10, 11]. The benefit of applying DL in radiomics is that it circumvents the suboptimal choice of generic features since a network can learn an optimal feature set specific to the task at hand, which could, in turn, could lead to higher predictive performance [12–15]. However, in practice, radiomic datasets often have very small sample sizes, which prevent the network from learning highly predictive features [16–18].

As an alternative, neural networks trained on data from other domains are used for feature extraction [19–21]. The intuition for using features adapted to another domain is based on the hope that they might also be informative when applied to radiomic data [22, 23]. In addition, these features could capture other aspects compared to generic ones, e.g., more global information [24]. The application of pretrained networks to radiomic data has other advantages as well. Most importantly, they may bypass the time-consuming fine segmentation of the pathologies often required to apply generic textural features; a simple volume of interest may be sufficient for a network to be predictive [25].

Therefore, one might assume that applying pretrained networks is simpler and more efficient than modeling using generic features. Unfortunately, to employ a pretrained network, several choices have to be considered, for example, the choice of network architecture and if and how to utilize fine segmentations. These choices are usually called hyperparameters and strongly affect the resulting model in general [26]. However, the impact of such choices on predictive performance in the radiomic context is unclear and has been studied only for specific datasets [27–29].

Accordingly, the goal of our study was to benchmark the effects of five choices, namely regarding the network architecture, the level of feature extraction, the use of segmentation, the number of used slices, and the type of feature aggregation, on the prediction performance using several radiomic datasets.

## Methods

### Ethical statement

Since only openly accessible and previously published datasets were used, ethical approval for this study was waived by the local Ethics Committee (Ethik-Kommission, Medizinische Fakultät der Universität Duisburg-Essen, Germany).

### Datasets

Ten publicly available datasets were used in this study (Table 1); six were taken from the “WORC” database [30], and the others were from The Cancer Imaging Archive (TCIA) [31]. Due to different reasons (e.g., missing or mismatching segmentation, too coarse slice thickness), a few scans have been removed from the datasets. More information is provided in Additional file 1.

**Table 1:** Datasets used in the experiments

Dataset	Modality (weighting)	*N*	In-plane resolution	Slice thickness	Source
C4KC-KiTS	CT	203	0.8 (0.4–1.0)	3.0 (1.0–5.0)	TCIA [14]
CRLM	CT	76	0.7 (0.6–0.9)	5.0 (1.0–8.0)	WORC [12]
Desmoid	MR (T1)	195	0.7 (0.2–1.8)	5.0 (1.0–10.0)	WORC [12]
GIST	CT	244	0.8 (0.6–1.0)	3.0 (0.6–6.0)	WORC [12]
HN	CT	134	1.0 (1.0–1.1)	3.0 (1.5–3.0)	TCIA [7]
ISPY-1	MR (DCE)	157	0.8 (0.4–1.2)	2.1 (1.5–3.4)	TCIA [15]
Lipo	MR (T1)	113	0.7 (0.2–1.4)	5.5 (1.0–9.1)	WORC [12]
Liver	MR (T2)	186	0.8 (0.6–1.6)	7.7 (1.0–11.0)	WORC [12]
Melanoma	CT	97	0.7 (0.5–1.0)	1.2 (0.6–2.0)	WORC [12]
TCGA-GBM	MR (T1)	53	0.8 (0.4–1.0)	5.0 (1.0–5.5)	TCIA [16]

For MR imaging, the used weighting is reported in parenthesis; N denotes the number of samples; and in-plane resolution and slice thickness are reported as median and range

### Study design

**Fig. 2:**
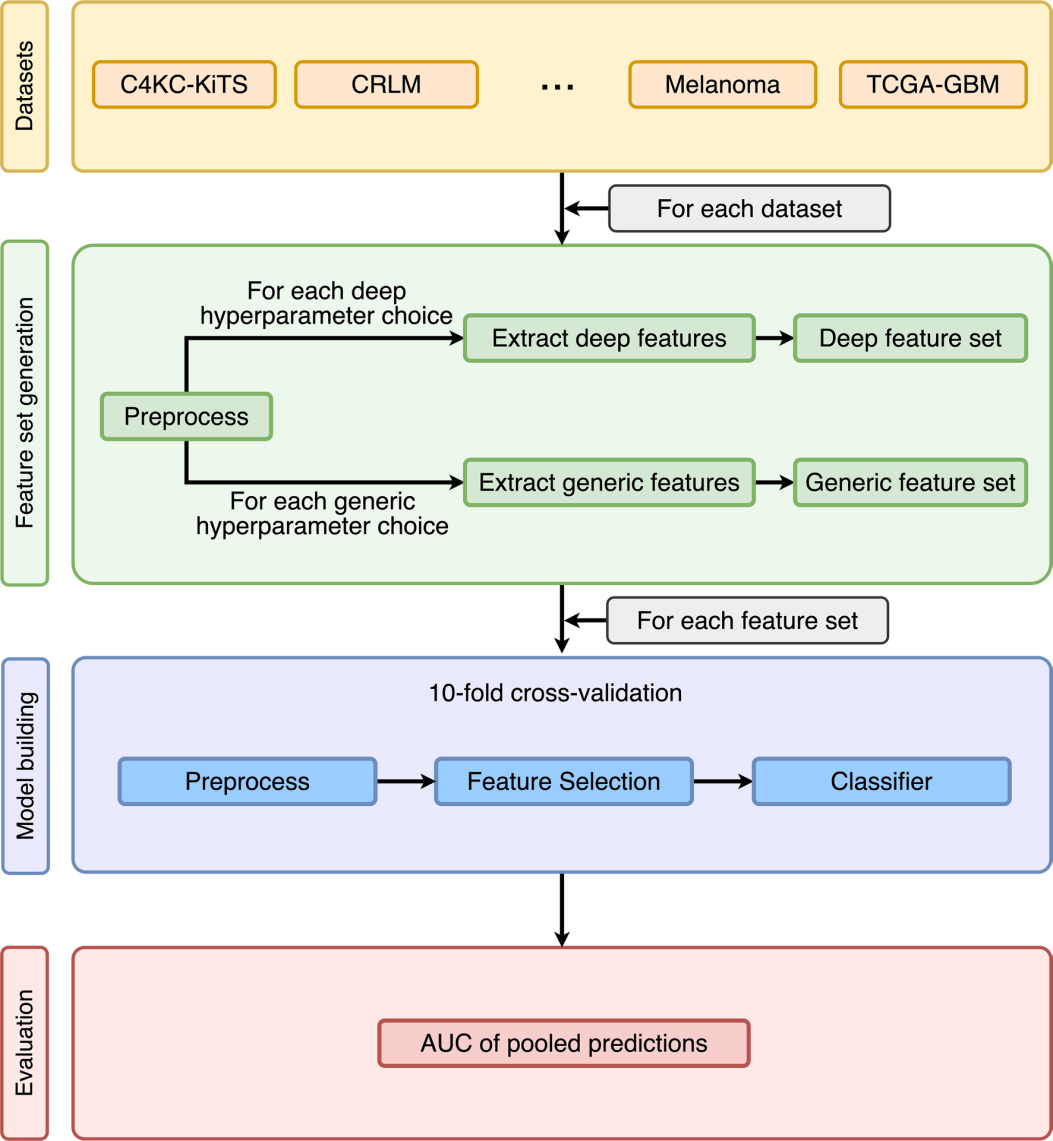
Flow diagram of the design of the study

The overall study design follows best practices in machine learning and can be seen in Fig. 2.

### Preprocessing

All scans were first isotropically resampled to a resolution of 1 mm^3^ using spline interpolation. Corresponding segmentations were interpolated using the nearest neighbor interpolation. MR images were first rescaled into the range 0–1, while for CT images, HU-values below -1024 and above 2048 were first set to zero before all values were rescaled into the range 0–1.

### Deep feature extraction

**Fig. 3:**
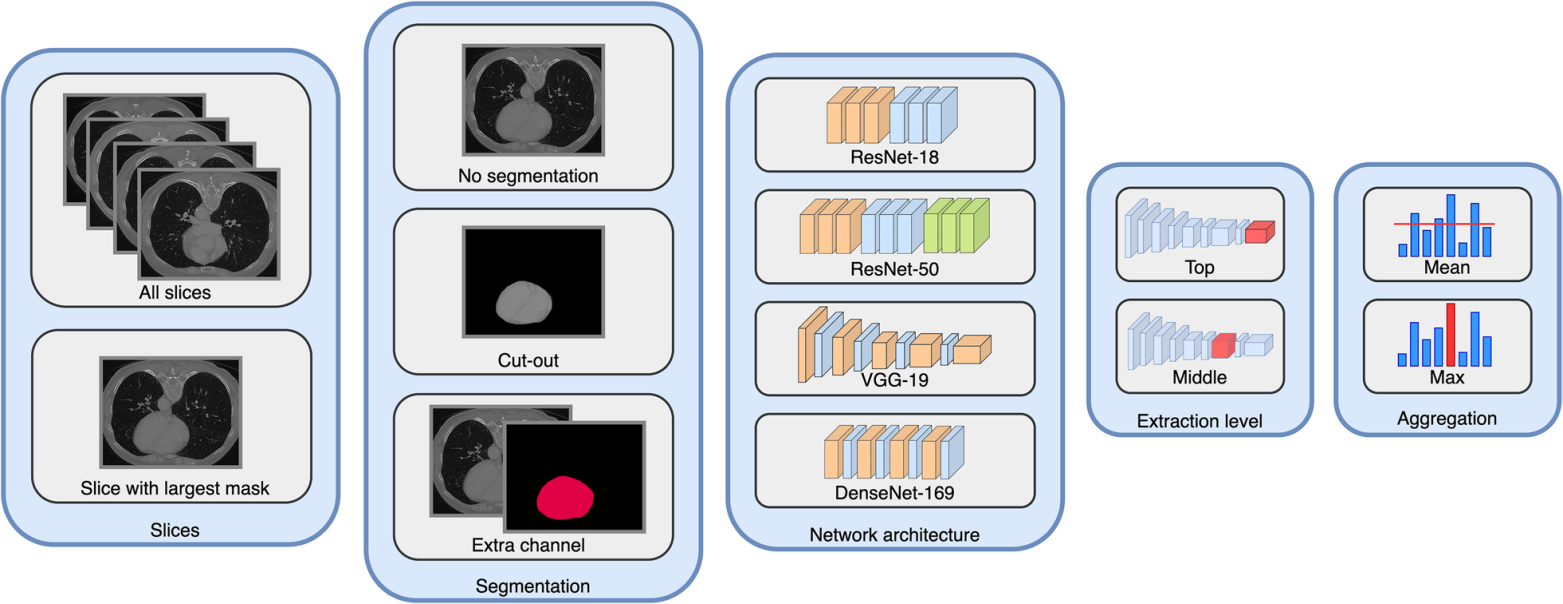
Graphical display of the choices for the extraction of deep features

Deep features were extracted slice by slice from the volume of interest (VOI) of a given scan (Fig. 3); here, the VOI is determined by the smallest bounding box around the segmentation. Since the slices were fed to pretrained networks trained on the ImageNet dataset, they were first rescaled to 224 × 224 pixels and then normalized so that their size, mean, and standard deviation fitted to those of the ImageNet dataset.

Extraction proceeded by feeding each scan slice to the selected network architecture, which outputted a feature vector. These feature vectors were then aggregated to form a single feature vector, which was inputted to the subsequent classifier.

There were several choices of how the extraction should proceed, which we treated as hyperparameters (Fig. 3): (A) whether to use all slices or only the slice with the largest (in-plane) segmentation; (B) whether to use the segmentation as an extra channel (ROI-channel), to use it to remove all pixels outside the area (ROI-cutout), or whether to ignore it (ROI-full); (C) which network architecture to use; (D) at which layer of the network to extract the features; and (E) how to aggregate the feature vectors of all extracted slices.

Altogether, we considered 96 = 4*2*3*2*2 different choices (Table 2) and thus 96 different feature sets. However, there is a dependency in these choices: If only the slice with the largest segmentation is selected, then the aggregation has no effect. Thus, in total, 72 different feature sets were considered.

**Table 2:** Hyperparameters for the extraction of deep features

Hyperparameter	Choices
Architecture	ResNet-18
	ResNet-50
	VGG-19
	DenseNet-169
Feature extraction level	Near-top
	Top
Segmentation	Only region of interest (ROI-full)
	Region of interest with mask as extra channel (ROI-channel)
	Cutout region of interest (ROI-cut)
Slices	Slice with largest segmentation area
	All slices
Aggregation	Maximum
	Mean

### Generic feature extraction

For comparison, generic feature sets were also extracted. Generic features depend on a discretization method in which the original intensity values are partitioned into bins [32]. Two different methods were considered: using a fixed bin width (of 10, 25, 50, and 100 units) and using a fixed bin count (with 10, 25, 50, and 100 bins). The extracted features comprised shape, first-order, gray-level co-occurrence matrix (GLCM), gray-level run length matrix (GLRLM), gray-level size zone matrix (GLSZM), neighboring gray-tone difference matrix (NGTDM), and gray-level dependence matrix (GLDM) features. All preprocessing filters were enabled, resulting in 2016 features for each patient. PyRadiomics 3.0.1 was used for extraction [33]. The complete list of features and other details can be found in Additional file 1 and the source code (https://github.com/aydindemircioglu/radPretrained).

### Feature preprocessing

Features were normalized by z-scores since it is well known that classifiers can be sensitive to different scales in the features. Constant features were removed from the dataset.

### Training

For modeling, six feature selection methods and five classifiers were employed. Hyperparameters were chosen from a prefixed set (Table 3). The feature selection methods provided a score for each feature that corresponded to its importance. Since it is not known beforehand which number of features works best, selecting 1, 2, 4, …, 64 features were tested.

**Table 3:** Overview of feature selection and classifier methods used

	Method	Hyperparameter
Feature selection	ANOVA	–
	Bhattacharyya distance	–
	Extra trees	Trees = 100
	LASSO	*C* = 1
	Random Forest	Trees = 100
	t-Score	–
Classifier	Logistic regression	*C* in 2^{− 6, − 4, − 2, 0, 2, 4, 6}
	Naive Bayes	–
	Neural network	Three layers with 4, 16 or 64 neurons each
	Random forest	Number of estimators 50, 125 or 250
	Support vector machines	*C* in 2^{− 6, − 4, − 2, 0, 2, 4, 6}, gamma was determined automatically

*C* denotes a hyperparameter regarding the regularization; higher *C* will make the model fit to the data more tightly

A stratified tenfold cross-validation was employed for training the models; the data were split into ten folds, and in each round, one fold was used for testing, while the other folds were used for training. Feature normalization, feature selection, and classifier training were processed only on the training fold [34]. The trained model was then applied to the test fold. These predictions were then pooled over all test folds to form a single receiver operating characteristics (ROC) curve, from which the area under the curve (AUC) was computed.

### Evaluation

First, the predictive performance of the models was evaluated. Since only the best model is essential from a practical point of view, the model with the highest AUC was selected for each parameter combination. A linear mixed model was then fitted to these data to measure the effects of the selected hyperparameters on the resulting AUCs. In the model, the dataset was treated as a random effect, which amounts to each dataset having its own baseline AUC; the influence of the parameters is then determined using this baseline value. In addition, the predictive performance was compared between the models based on deep and generic features using a DeLong test. Calibration curves were also plotted to judge the quality of the predictions.

Second, correlations between deep and generic feature sets were calculated to measure the extent to which they are capture similar information. For this, the average Pearson correlation between each pair of features was computed.

*p* values below 0.05 were considered to be statistically significant. Results were corrected for multiple testing using the method of Holm. The mixed model was computed using Python 3.8 and the statsmodel package.

## Results

Overall, 80 feature sets (72 deep and 8 generic) were extracted for each of the seven datasets.

### Predictive performance

Considering the best-performing models (Table 4), no clear pattern in the parameters of the deep models could be seen, although models ignoring the fine segmentation (ROI) were performing best on only one dataset. The same is valid to some extent for models using generic features; here, models using bin count as a discretization method were less likely to perform best.

**Table 4:** AUCs of the best-performing models for each dataset

Dataset	Parameters of deep model					AUC (deep features)	Parameter of generic model			
	Architecture	Extraction level	Slices	Aggregation	Segmentation		Discretization	AUC (generic features)	*P* (deep vs generic)	AUC (from other studies)
C4KC-KiTS	ResNet-50	Mid	All	Max	ROIchannel	0.75	binWidth:25	0.76	− 0.01 (*p* = 0.716)	0.88 [18]
CRLM	ResNet-18	Top	Max	Mean	ROIchannel	0.81	binWidth:10	0.79	0.02 (*p* = 0.715)	0.68 (0.56–0.8) [19]
Desmoid	ResNet-50	Mid	All	Max	ROI	0.86	binWidth:10	0.89	− 0.03 (*p* = 0.456)	0.82 (0.75–0.89) [19]
GIST	VGG-19	Mid	Max	Mean	ROIchannel	0.79	binWidth:25	0.78	0.01 (*p* = 0.674)	0.77 (0.71–0.83) [19]
HN	VGG-19	Mid	All	Mean	ROIchannel	0.91	binCount:50	0.89	0.02 (*p* = 0.405)	0.84 (0.77–0.91) [19]
ISPY-1	VGG-19	Mid	All	Mean	ROIchannel	0.78	binWidth:25	0.7	0.08 (*p* = 0.109)	–
Lipo	ResNet-18	Top	All	Mean	ROIcut	0.92	binWidth:25	0.9	0.02 (*p* = 0.535)	0.83 (0.75–0.91) [19]
Liver	DenseNet169	Top	All	Max	ROIcut	0.82	binWidth:100	0.79	0.03 (*p* = 0.526)	0.81 (0.75–0.87) [19]
Melanoma	DenseNet169	Mid	All	Mean	ROIcut	0.82	binCount:50	0.72	0.1 (*p* = 0.176)	0.51 (0.4–0.62) [19]
TCGA-GBM	ResNet-50	Mid	Max	Mean	ROIcut	0.89	binWidth:25	0.79	0.1 (*p* = 0.176)	–

AUC of the best-performing models for each dataset. Statistical difference was tested with a DeLong test. AUCs (with 95% CI where applicable) from other studies were reported in the last column; for ISPY-1 and TCGA-GBM, no corresponding studies using only a single MR-weighting were found

These best-performing models were used to fit a mixed linear model; because 10 datasets and 72 feature sets per dataset were employed, overall, 10*72 = 720 data points were utilized in the regression. In the model, all factors were statistically significant (Table 5), except for the aggregation of feature vectors, where no difference was found between using the mean or the maximum value (*p* = 0.774). However, using the ResNet-50 architecture instead of DenseNet-169 resulted in a statistically significant effect of + 0.016 in AUC (*p* < 0.001), while no significance was reached when using ResNet-18 or VGG-19. Regarding the feature extraction level, using features from a level below the top level was beneficial (AUC + 0.016; *p* < 0.001). Concerning the segmentation, not using it was no different from using it to mask intensity values (*p* = 0.455). However, adding them as an additional channel was slightly helpful (AUC + 0.011; *p* = 0.023). In addition, using only the slice with the maximum segmentation area instead of all slices produced worse results (AUC − 0.023; *p* < 0.001).

**Table 5:** Results of the mixed linear model

Parameter		Estimate	Confidence interval	*p* value
Fixed effects				
Architecture	DenseNet-169	(Baseline)		
	ResNet-18	0.004	− 0.004; 0.012	0.647
	ResNet-50	0.016	0.008; 0.024	< 0.001
	VGG-19	0.01	0.002; 0.018	0.096
Extraction level	Near-top	(Baseline)		
	Top	− 0.016	− 0.021; − 0.01	< 0.001
Segmentation	ROI	(Baseline)		
	ROIchannel	0.011	0.003; 0.018	0.023
	ROIcut	0.005	− 0.002; 0.012	0.455
Slices	All	(Baseline)		
	Mean	− 0.023	− 0.03; − 0.016	< 0.001
Aggregation	Max	(Baseline)		
	Mean	0.001	− 0.006; 0.008	0.774
Intercept		0.741	0.703; 0.78	< 0.001
Random effects				
Dataset (variance)		0.002		< 0.001

For the mixed linear model, the dataset was considered to be a random effect. Only the best-performing models measured in AUC were included in the mixed model. p values were corrected for multiple testing using the Holm method

Differences in AUC could be seen when comparing the best-performing model based on deep features with the best-performing model using generic features (Table 4). All absolute differences were smaller than 0.03, except on three datasets (ISPY-1, Melanoma, and TCGA-GBM), where the difference was 0.08–0.10. However, on all datasets, the differences in AUC were not significant when compared with a DeLong test. Regarding the calibration curves (Additional file 1: Fig. S1), the models are rather calibrated except for CRLM and ISPY-1.

### Correlations

**Fig. 4:**
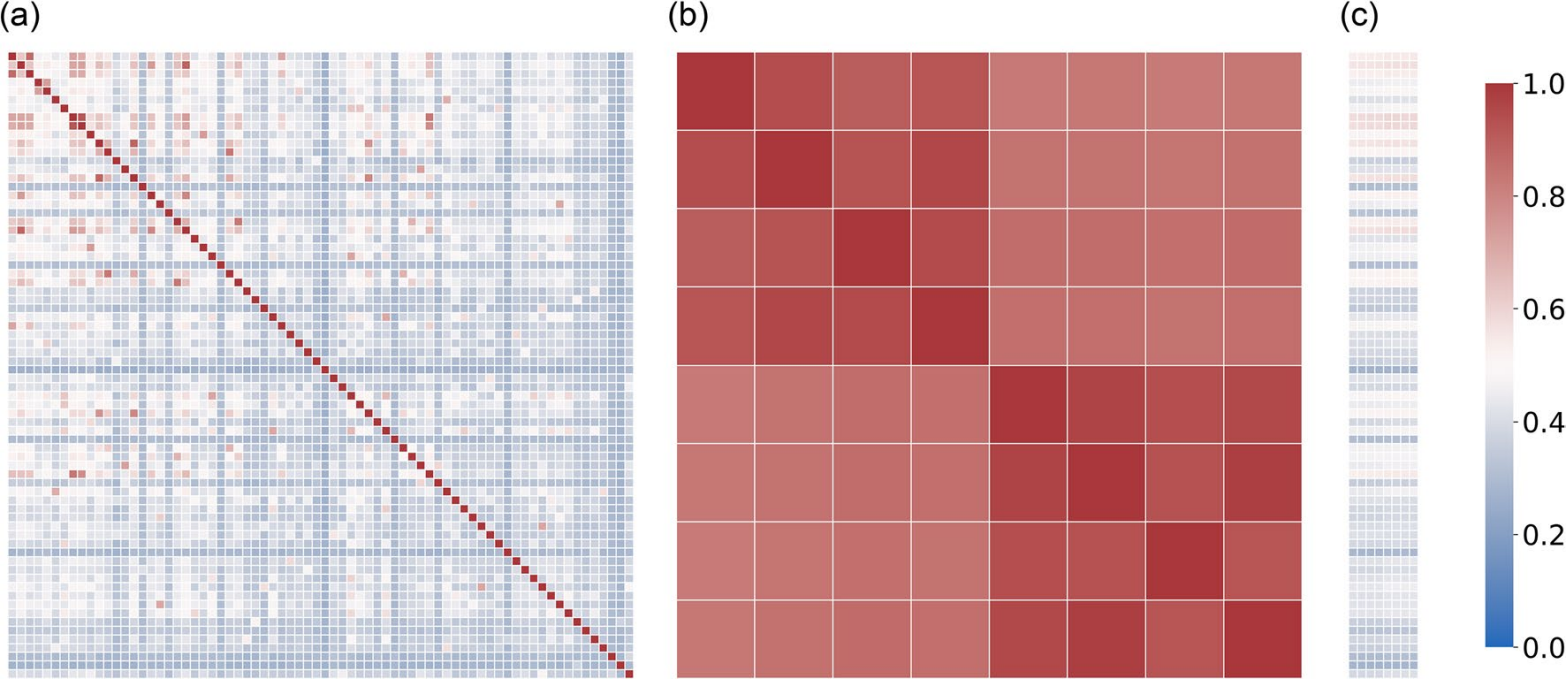
Graphical display of the correlations between all feature sets. Mean pairwise Pearson correlation between (**a**) deep, (**b**) generic, (**c**) deep and generic feature sets. Red colors correspond to higher correlations and blue colors to lower. The mapping is the same for all three figures and is displayed in the legend at the right

The mean correlations between the different deep feature sets were moderate on average, *r* = 0.4, varying between *r* = 0.25 and *r* = 0.92 (Fig. 4a). For the generic feature sets, the correlation was much higher, *r* = 0.89, and varied between 0.83 and 0.98 (Fig. 4b). When comparing generic and deep features, the correlation was moderate, *r* = 0.43, and varied between 0.25 and 0.66 (Fig. 4c).

## Discussion

Deep features extracted from pretrained networks have been used in radiomics for several reasons; however, no systematic study of the impact of these choices on different radiomics datasets has been performed yet. Our study investigated different choices for deep feature generation from a practical perspective using ten publicly available datasets and demonstrated that these choices have a large impact on the predictive performance of the resulting feature sets.

Yet, when considering the best-performing models, no clear pattern emerged. For example, using features from the top layer of the networks yielded the best-performing model for three of the datasets. This shows that it is not a single model that gives the best results but that the feature extraction parameters must be optimized during cross-validation to achieve the best performance.

To determine statistically which of the choices had a significant influence on the predictive performance, we employed a mixed linear model. In this model, the most influential parameter was whether to use all the slices or only the one with the largest mask (AUC − 0.023; *p* < 0.001). This finding is not unexpected since using only the slice with the largest mask potentially disregards much information, especially concerning the spatial structure of the pathology.

Next, the network architecture had the largest impact (AUC + 0.016; *p* < 0.001). While there was no statistically significant difference between the DenseNet-169, the ResNet-18, and the VGG-19, the ResNet-50 performed better. This is partly surprising because no training was performed. We suspect this might be related to the network size: Larger networks extract features that might be too fine-grained for the radiomic context while smaller networks might extract too coarse features. The medium size of the ResNet-50 seems to be a good balance.

Furthermore, extracting features from a level below the top level was beneficial (AUC 0.016; *p* < 0.001). One reason could be that the features at the top level are more adapted to the training set on which the pretraining was performed. In contrast, features from a lower level could be more general and thus more helpful in the radiological context.

Regarding the segmentation, no considerable difference was seen between ignoring it and using it to cut out the region of interest (AUC + 0.005; *p* = 0.455); however, adding the segmentations as another channel was slightly beneficial (AUC + 0.011; *p* = 0.023). This difference could indicate that the peritumoral region has some information that the deep network can use, which has already been observed in some studies [35, 36]. However, it is unclear how fine the pathology must be delineated; in a few cases, a rough outline can work well [37], while in others, it might not be feasible [38].

Finally, aggregating the information into multiple slices using the feature-wise maximum did not differ from using the feature-wise average (AUC + 0.001; *p* = 0.774).

Therefore, considering all these observations, it should be beneficial to use a medium-scale network such as the ResNet-50 with features extracted from a level below the last convolutional layer. All slices should be processed, and segmentations should be added as an additional channel.

Surprisingly, deep features did not outperform general modeling consistently in our study. While the overall AUCs are generally higher (except for C4KC-KiTS), the difference was always not statistically significant. However, two things should be kept in mind: The overall sample sizes were relatively small, and the pretrained deep networks were based on single slices. It is conceivable that 3-D networks with larger sample sizes will perform better.

Regarding the correlation among the feature sets, some correlations were very low (*r* = 0.25), while some were very high (*r* = 0.92). It shows that the different decisions to extract the deep features greatly impacted the resulting features. Similarly, comparing the correlation between the generic and the deep features, there were sets with low (*r* = 0.25) and moderate correlations (*r* = 0.66). Therefore, it is reasonable to expect the feature sets to be relatively different and capture different information. Finally, the correlations among the generic feature sets were quite large, and correlations between 0.83 and 0.98 were seen. In other words, the influence of the choice of bin width and bin counts on the resulting dataset is much lower than using deep features.

Our study has limitations. For one, it is limited by the fact that only cross-validation was used. However, the sample sizes of radiomics datasets make it hard to split off a reasonably sized hold-out set, and external validation sets are unavailable for the datasets we used. Because of this, we cannot rule out that overfitting might have occurred in our study. Yet, for the WORC datasets, a comparison to the results of the study by Starmans et al. [39] shows that the AUCs we obtained from the deep networks are very well within the 95% confidence interval (CI) they have stated. A large discrepancy was seen in the Melanoma dataset. Starmans et al. state that they failed to build a predictive model for the melanoma dataset and argue that this was a good thing since physicians could also not predict the BRAF mutation staging. Therefore, our result may indicate overfitting. However, this is not the only explanation since radiomics models are thought to exploit the structures in given data better than humans [2]; only with an independent validation set can a decision be made in this regard.

In addition, we only considered the predictive performance, although many other aspects play a crucial role, especially for application in clinical routine. This encompasses the reproducibility of the features, which in turn depends on the imaging protocols and scanning hardware. It is also well known that there is a significant effect on the features stemming from the intra- and inter-variability of the segmentations [40–42]. Unfortunately, analyzing these aspects would require corresponding datasets, which are currently not openly available.

Furthermore, the predictive performance for a single dataset can have more hyperparameters than considered here. For example, multiparametric MRI or PET-CT needs another aggregation step regarding the different modalities. We also restricted ourselves to pretrained 2-D networks since end-to-end modeling with low sample sizes might not be feasible and considered five different options for extracting features from these. In practice, numerous other techniques are used to increase predictive performance; for example, augmentations (at train time and test time) can partly be used to circumvent the problem of low sample sizes. These points should be taken into consideration in future studies.

## Conclusions

Our study demonstrated that deep feature sets depend significantly on the choices regarding the extraction from the pretrained deep network. These choices should ideally be optimized for obtaining the best-performing model during cross-validation. Nonetheless, we could not find a significant increase in the predictive performance of these models compared to models trained on generic features.

## Supplementary Information

**Additional file 1.** Details on the training procedure and the resulting calibration curves.

## Data Availability

The datasets supporting the conclusions of this article are available in the GitHub repository (https://github.com/aydindemircioglu/radPretrained).

## Abbreviations

ANOVA: Analysis of variance
AUC: Area under the curve
CI: Confidence interval
CNN: Convolutional neural network
CT: Computed tomography
CV: Cross-validation
DCE: Dynamic contrast-enhanced
LASSO: Least absolute shrinkage and selection operator
MR: Magnetic resonance
RBF-SVM: Support vector machine with radial basis function kernel
RF: Random forests
ROI: Region of interest; SVM: Support vector machine
TCIA: The Cancer Imaging Archive
