# Supplementary material for: Predictive performance of radiomic models based on features extracted from pretrained deep networks
Source: Insights Imaging. 2022 Dec 9;13:187. doi: 10.1186/s13244-022-01328-y (PMC9733744; doi:10.1186/s13244-022-01328-y)
Supplement: Supplementary file 1 — Additional file 1. Details on the training procedure and the resulting calibration curves. [file 13244_2022_1328_MOESM1_ESM.pdf]

# ELECTRONIC SUPPLEMENTARY MATERIAL

## Predictive performance of radiomic models based on features extracted from pretrained deep networks

### *Datasets*

All datasets were resampled to 1x1x1 $cm^3$, regardless their original spacing. A few samples were removed due to different errors (< 10 scans for each dataset), e.g., if the slice thickness was much larger than the median, or if PyRadiomics issued an error. The full list of excluded scans can be found in the source code (./data/blacklist.csv).

**ISPY-1:** The processed data and segmentations by Chitalia et al. was used (https://doi.org/10.7937/TCIA.XC7A-QT20). The second dynamics was taken as series (ending with DCE_0001.dcm).

**GBM:** The processed data and segmentations by Beers et al. was used (https://doi.org/10.7937/TCIA.2018.ow6ce3ml). The T1-weighting was used as series. Labels were taken from (https://github.com/JSB-UCLA/ITCA).

**C4KC-KiTS:** Labels were taken from https://github.com/wukaiyeah/kidney_cancer_project

**WORC:** The python loader script from the WORC dataset was used to download all images and segmentation.

### *Slice extraction*

From the segmentation, a volume of interest was created by taking the smallest bounding around the segmentation. The slices from CT scans were normalized by setting all values below

-1024 to -1024 and all values larger than 2048 to 2048. Then the intensity was shifted by 1024 so that no negative values existed and divided by 3072 (the range) to obtain values between 0 and 1. Then, values were multiplied by 255 and converted to integers. Slices from the MR were linearly normalized to the range 0-255.

Slices where the segmentation was too small (less pixel than a circle with a diameter of 5 mm) were not considered.

In the case of ROI-channel, the first channel was the original image slice, while the second was the binary segmentation mask. The third channel was a simple linear mean of both channels. For ROI-cutout, the image was multiplied with the segmentation mask and was duplicated to obtain an RGB image. Finally, for ROI-full the segmentation is ignored, and the input image is converted to RGB by repeating the channel.

**Feature extraction**

Features were extracted from each network's top or below the top layer. In more detail, using the notation of the models from torchvision; for the ResNet18, the top features were extracted from the "flatten" layer and the near-to-top features from "layer3.1.relu_1" layer. For ResNet50, the top features were extracted from the "flatten" layer, and the near-to-top features from "layer3.5.relu_2" layer. For the DenseNet169, the top layer was "flatten", the near-to-top layer the "features.transition3.pool" layer. Finally, for the VGG19 network, the top layer was "feature.35", while the near-to-top layer was "features.26".

## Calibration curves for the best-performing model

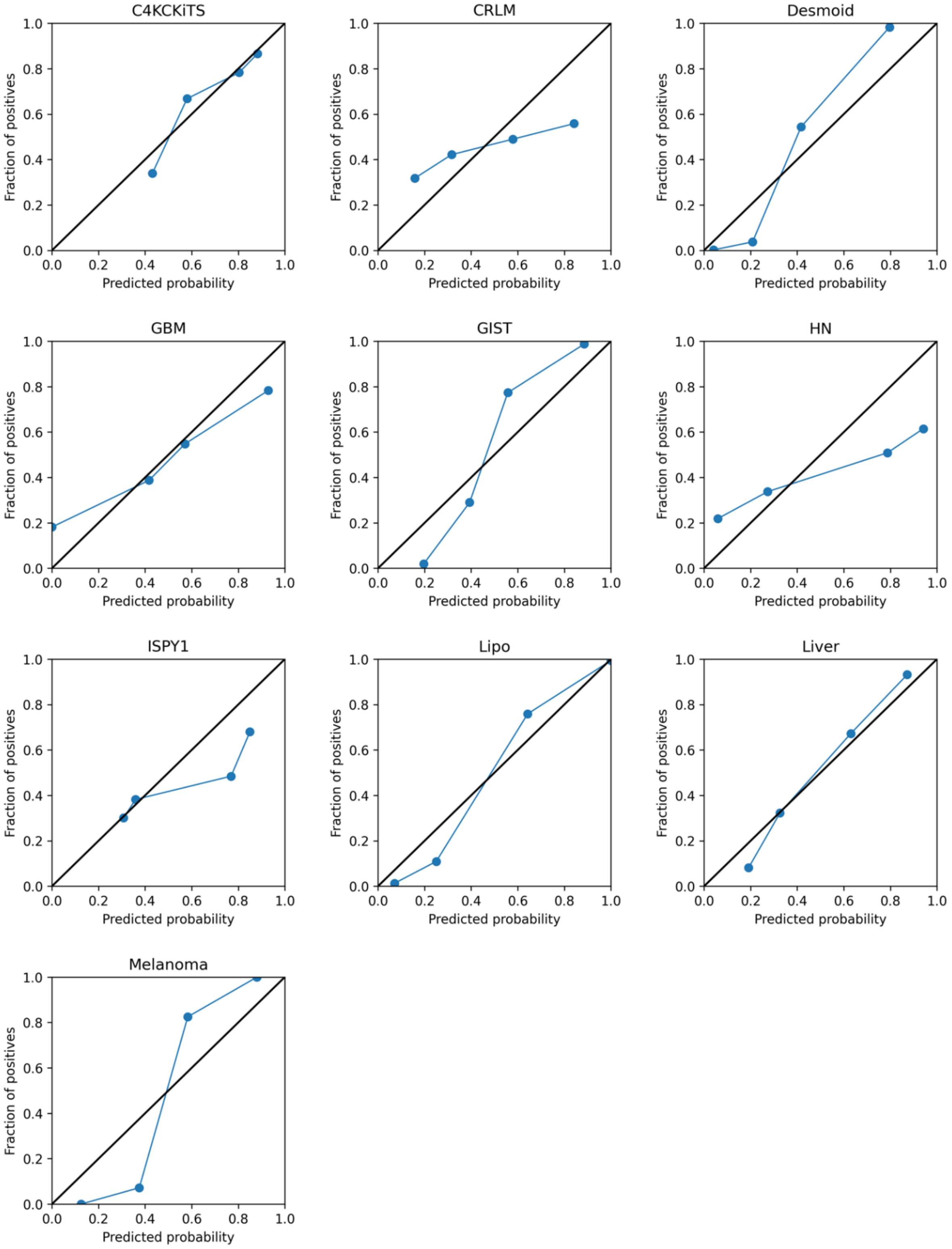


**Figure S1:** Calibration curves of the best-performing models.

**Source code**

The source code of the experiments along with the results can be found in

http://github.com/aydindemircioglu/radPretrained .
